# Comparative Analysis of Laparoscopic Cholecystectomy Performed in the Elderly and Younger Patients: Should We Abstain from Laparoscopic Cholecystectomy in the Elderly?

**DOI:** 10.7759/cureus.2888

**Published:** 2018-06-27

**Authors:** Ugur Ekici, Serhan Yılmaz, Faik Tatlı

**Affiliations:** 1 Health Science and Administratioon, İstanbul Gelisim University, İstanbul, TUR; 2 General Surgery, Bakirkoy Sadi Konuk Education and Research Hospital, İstanbul, TUR; 3 General Surgery, Harran, Sanlıurfa, TUR

**Keywords:** laparoscopic cholecystectomy, cholelithiazis, senility, complication, mortality, morbidity, gall bladder, conversion to open

## Abstract

Background: The elderly population is gradually increasing due to an increase in the quality of life and therefore the frequency of gallbladder stones in the population is also increasing. However, a considerable number of physicians tend to postpone or solve the problem with medical treatment instead of performing surgery in the elderly patients. In this study, we aim to compare the outcomes of laparoscopic cholecystectomy (LC) in the elderly and younger patients.

Material and Methods: The medical records of 665 patients undergoing LC were evaluated retrospectively. The patients were divided into two groups: ≥60 years of age and <60 years of age. Ages, genders, comorbid diseases, indications of surgery, American Society of Anesthesiologists scores, whether it is converted to an open cholecystectomy or not, reasons for conversion if it is converted, total duration of surgery, initiation of oral nutrition, duration of discharge, and postoperative complications of the patients in both groups were recorded.

Results: The American Society of Anesthesiologists scores were statistically significantly higher in ≥60 years age group (p<0.001). The rate of experiencing acute cholecystitis with a stone in the gallbladder was significantly higher in the 60 years group (p=0.025). Comorbidity was statistically significantly higher in the ≥60 years age group (p<0.001). Hospitalization period, the mean hour of initiation of oral nutrition were statistically significantly higher in the ≥60 years age group (p<0.001, p=0.001). Conversion to an open cholecystectomy and postoperative complication rates of the ≥60 years age group were statistically significantly higher (p=0.034, p<0.001).

Conclusion: We think that LC can be safely performed in the elderly people as well. However, it should be kept in mind that comorbidity may make the surgery and postoperative follow-up period complicated.

## Introduction

Cholelithiasis is considered to be the most frequent gallbladder disease. Its prevalence is below 5% among <40-year-old individuals, whereas the prevalence increased up to 30% among individuals aged more than 80 years [1]. The golden standard in the treatment of cholelithiasis is laparoscopic cholecystectomy (LC). LC is more advantageous since it offers less pain, earlier discharge, early recovery, better cosmetic outcomes, and low cost [2]. Due to an increase in the quality of life, the elderly population gradually increases and hence, the prevalence of cholecystitis in the population also increases. The age-dependent comorbidities that might arise are the most important factors increasing the possibility of postoperative mortality and morbidity [3-4].

In the present study, we aim to compare the outcomes of LC operation among elderly and young patients.

## Materials and methods

The medical records of 665 patients undergoing LC between May 2013 and May 2017 were evaluated retrospectively. The patients were divided into two groups as ≥60 years of age and <60 years of age. Ages, genders, comorbid diseases, indications of surgery, American Society of Anesthesiologists (ASA) scores, whether it is converted to an open cholecystectomy or not, reasons for conversion if it is converted, total duration of surgery, initiation duration of oral nutrition, duration of discharge and postoperative complications of the patients in both groups were recorded. Abdominal ultrasonography was performed in all of the patients before surgery. The surgery was performed in patients with a choledochus duct stone after magnetic resonance cholangiography (MRCP) as well as endoscopic retrograde cholangiopancreatography (ERCP) if required. The patients undergoing surgery due to acute cholecystitis were operated within the first 72 hours. Medical treatment was administered to the patients presenting later than this period and they were operated after eight weeks. One gram of sefazolin sodium was administered preoperatively in the patients. Surgeries were performed by four surgeons. Surgery was started with the laparoscopic method in all of the patients. The standard four-trocar method was used. The mean follow-up time of the patients was 26 (range: 5-48) months. Redness, elevated temperature, and the presence of purulent discharge at the site of trocar was considered to be positive for wound site infection.

SPSS 15.0 (SPSS Inc., Chicago, IL, USA) program was used for statistical analysis. Descriptive statistical methods were expressed as numbers and percentages for categorical variables and expressed as mean, standard deviation, minimum and maximum for numerical variables. Since numerical variables did not meet the condition of normal distribution in both the independent groups, Mann-Whitney U test was used for the comparison. The rate of categorical variables between the groups was analyzed by using Chi-square analysis. In cases where the conditions were not met, Monte Carlo simulation was performed. Statistical significance level (alpha) was considered to be p<0.05.

## Results

The mean age of 665 patients included in the study was 45.8 (16-84) years. One hundred and twenty-seven (19.1%) of them were in ≥60 years age group and 538 (80.9%) of them were in <60 years age group. Five hundred and twenty-two (78.5%) of them were females and 143 (21.5%) of them were males. Five hundred and seventy-nine patients (87.1%) had cholelithiasis. Choledocholithiasis was determined in eight patients before surgery. The stone was removed by performing ERCP after MRCP and the patients were operated within a mean of four weeks. Sixty-four (9.6%) patients were operated while they were in the period of acute cholecystitis, 117 (17.6%) patients were operated after an eight-week waiting period following medical treatment.

There was no statistically significant difference between the gender rates of ≥60 years of age patients and <60 years of age patients (p=0.579). The rate of surgery due to acute cholecystitis with a stone in the gallbladder was higher in the ≥60 years age group (p<0.001). The rate of being symptomatic and experiencing a cholecystitis attack previously was significantly higher in the ≥60 years age group when compared to the <60 years age group (p=0.007, p=0.025; respectively) (Table 1).

**Table 1:** Preoperative status n: number; ACC: acute calculous cholecystitis; AAC: acute acalculous cholecystitis; *: statistically significant.

Demografical situation	<60 year-old	≥60 year-old	
	n (%)	n (%)	p
Gender			
Female	420 (78.1)	102(80.3)	0.579
Male	118 (21.9)	25 (19.7)	
Reason for surgery			
Cholelitiazis	478 (88.8)	101(79.5)	0.002^*^
Polyp	15 (2.8)	1 (0.8)	0.331
A.C.C	41 (7.6)	23 (18.1)	<0.001^*^
A.A.C	2 (0.4)	1 (0.8)	0.471
Adenomyomatozis	2 (0.4)	1 (0.8)	0.471
Symptomatic	406 (75.5)	110(86.6)	0.007^*^
History of acute cholecystitis attack	86 (16)	31(24.4)	0.025^*^

The majority of the patients had an American Society of Anesthesiologists (ASA) score of I and II: 463 (69.6%), 184 (27.7%); respectively. The most common comorbid diseases were hypertension (HT) 110 (16.5%); diabetes mellitus (DM) 61 (9.2%); chronic obstructive pulmonary disease (COPD) 53 (8%); and (4.1%) congestive heart failure (CHF). There was a statistically significant difference between ASA score rates of groups (p<0.001). ASA I (27 patients) and ASA II patients were statistically significantly higher in the <60 years age group and ≥60 years age group, respectively (p<0.001). The rate of comorbid diseases of the ≥60 years age group was statistically significantly higher compared as to the <60 years age group (p<0.001). Among comorbid diseases, the rate of DM, HT, COPD, CHF was statistically significantly higher in the ≥60 years age group as compared to the <60 years age group (p<0.001 for all of them) (Table 2).

**Table 2:** ASA scores and comorbidity n: number; ASA: American Society of Anaesthesiologists; DM: diabetes mellitus; HT: hypertension; COPD: chronic obstructive pulmonary disease; CCF: congestive cardiac failure; *: statistically significant.

	<60 year-old	≥60 year-old	
	n (%)	n (%)	p
ASA Scores			
1	433 (80.5)	30 (23.6)	<0.001^*^
2	100 (18.6)	84 (66.1)	<0.001^*^
3	4 (0.7)	10 (7.9)	<0.001^*^
4	1 (0.2)	3 (2.4)	
Co-morbitity presence	98 (18.2)	98 (77.2)	<0.001^*^
DM	31 (5.8)	30 (23.6)	<0.001^*^
HT	53 (9.9)	57 (44.9)	<0.001^*^
COPD	20 (3.7)	33 (26)	<0.001^*^
CCF	8 (1.5)	19(15)	<0.001^*^
Others	7 (1.4)	7 (5.6)	0.244

The surgery was converted to an open cholecystectomy in 37 patients (5.6%). Twenty-five patients (4.6%) in the <60 years age group and 12 patients (10.4%) in the ≥60 years age group underwent an open cholecystectomy. The risk for conversion to an open cholecystectomy increased more than two-fold in the ≥60 years age group, and conversion to an open cholecystectomy was statistically significantly higher in the ≥60 years age group as compared to the <60 years age group (p=0.034). The most common reason for conversion to an open cholecystectomy was the uncertainty of the structures within the Calot's triangle, preventing a safe dissection in 15 patients (40%). Besides, the cause for conversion to an open cholecystectomy in patients was adhesion in 13 (35%) patients, uncontrolled bleeding in six (16.2%) patients, bile duct injury in two (5.4%) patients, and gallbladder tumour in one (2.7%) patient. Mean duration of surgery was 45.8 (±18,3) minutes. A drain was inserted in 187 patients (28.1%) for the purpose of follow-up. Duration of drain removal was mean 1.7 days. Hospitalization period and the mean hour of initiation of oral nutrition were statistically significantly higher in the ≥60 years age group as compared to the <60 years age group (p<0.001, p=0.001), respectively (Table 3).

**Table 3:** Surgery type and postoperative period N: number; *: statistically significant.

		<60 year-old		≥60 year-old		
		Mean ±SD	Median	Mean ±SD	Median	p
Hospitalization duration (days)		1,08±0,46	1	1,28±0,79	1	0,001*
Time to begin oral feeding (hours)		6,25±3,91	6	7,20±5,08	6	0,001*
Duration of operation (minutes)		52,5±16,1	50	57,1±21,2	55	0,053
		n	%	n	%	p
Type of surgery	Laparoscopic	513	95,4	115	89,6	0,187
	Convertion	25	4,6	12	10,4	0,034^*^

Postoperative complications developed in 47 (7.1%) patients. The frequency of postoperative complication and pulmonary complication among these complications was statistically significantly higher in the ≥60 years age group compared to the <60 years age group (p<0.001, p=0.001), respectively (Table 4). One of the two patients was a 50-year-old ASA IV patient who developed sepsis and had multiple organ failures due to acalculous cholecystitis. The other patient was an 84-year-old ASA IV patient undergoing surgery due to acute cholecystitis with a stone in the gallbladder and developing sepsis.

**Table 4:** Postoperative complications n: number; *: statistically significant.

	<60 year-old	≥60 year-old	
	n (%)	n (%)	p
Postoperative Complication presence	26 (4,8)	21 (16,5)	<0,001*
Wound site infection	14 (2,6)	5 (3,9)	0,383
Hemorrhage	1 (0,2)	2 (1,6)	0,095
Biliary tract injury	2 (0,4)	3 (2,4)	0,051
Thrombophlebitis	1 (0,2)	0	1,000
Urinary Infection	3 (0,6)	3 (2,4)	0,087
Pulmonary Complication	0	4 (3,1)	0,001*
Incisional hernia	2 (0,4)	0	1,000
Choledocal stone	2 (0,4)	3 (2,4)	0,051
Exitus	1 (0,2)	1 (0,8)	0,346

## Discussion

For the developing nations, the World Health Organization (WHO) has defined the elderly as people aged 60 or older and 65 and over for developed nations [5]. Together with the advancements in medical care, there has been an increase in geriatric diseases among the aging population. The most frequent surgical disease among the elderly individuals is cholelithiasis [6]. Although advanced age is not considered as a contraindication for surgical treatment, a majority of the physicians tend to delay the surgical operation or preferring medical treatment rather than surgical intervention for the elderly patients [7]. The golden standard in cholecystectomy operations is to employ the laparoscopic method in the operation.

Conversion to open cholecystectomy is required when LC cannot be safely continued and/or there are intraoperative complications. The mean rates of conversion from LC to open cholecystectomy were reported in male and female patients as 5.6% and 2.2%, respectively [8]. The value of the same parameter was calculated to be 5.6% for our patients. The most frequent reason for conversion to open method is the inability of identifying the anatomic structures and thus the impossibility of safe dissection [9-10]. Among 37 (5.6%) patients for whom we converted to open method, the inability of identifying the anatomic structures played an effective role in 15 cases (40%). The advanced age was reported to be a risk factor for conversion to open method [11]. In our patient groups, this risk was statistically significantly higher in the ≥60-year-old group (p=0.034).

With advanced age, the incidence of cholelithiasis also increases and thus the incidence of acute cholecystitis also increases among the elderly patients. As a result, the complications become more frequent [12]. Annually 1%-4% of patients with gallbladder stone became symptomatic; majority of these symptoms is the biliary colic, whereas acute cholecystitis develops in many of the cases [13]. In the present study, the number of patients that have a history of acute calculous cholecystitis attack (p=0.025) and surgery due to acute cholecystitis (p<0.001) was statistically significantly higher in the ≥60-year-old group.

In the treatment of acute cholecystitis, it is not clear whether the best option is early or delayed cholecystectomy [13]. Gruamsy et al., in their meta-analysis, reported that there was no difference between early and delayed surgery in treatment of acute cholecystitis in terms of mortality and morbidity [14] whereas Cao et al. reported in their meta-analysis that early surgery is superior to delayed surgery in terms of mortality and morbidity, and they recommended early surgery [15]. In the present study, LC was performed within first 72 hours for the acute cholecystitis patients. The patients exceeding beyond this time limit were given medical treatment and then underwent LC after an eight-week waiting period.

LC is considered as a safe method for elderly patients as well. However, there are studies reporting higher hospitalization duration, mortality and morbidity rates for patients with higher ASA scores [8-16]. In a study by Yi et al., the authors reported higher ASA score to increase the mortality and morbidity but not affect the operation duration and time to discharge [17]. The morbidity among elderly patients who had undergone LC was reported to be 5%-15%, whereas mortality was reported to be 0%-1% for the same group [18-19]. In the present study, the morbidity of elderly patients was 15.7%, while mortality was calculated to be 0.8%. The morbidity was statistically significantly higher in the ≥60-year-old group (p<0.001). The hospitalization duration (p<0.001) and time to begin oral feeding (p=0.001) were statistically significantly higher in the ≥60-year-old group. Among the postoperative complications, the lung complications were statistically significantly higher (p=0.001). The most frequently seen complication was observed to be the wound site infection seen in 19 cases (2.7%). The wound site infection is related to acute cholecystitis and DM [20-21]. But, although the incidence of acute cholecystitis and DM was statistically significantly higher in the ≥60-year-old group in the present study, there wasn’t a statistically significant difference in terms of wound site infection.

The most frequently seen and feared postoperative complication observed after LC was reported to be the biliary tract injury [22-23]. Although the hemorrhage was reported rarely, it might be at a life-threatening level [22]. Although the increase in safe implementation of LC is believed to reduce the rate and the incidence of biliary tract injury following cholecystectomy (reported to be 0.5%-1.4%), the incidence of hemorrhage was reported to be 1%-2% [23-24]. In the present study, the biliary tract injury was observed in five cases (0.8%), while hemorrhage developed in three cases (0.5%). Three (2.4%) of the patients with biliary tract injury and two (1.6%) of the patients with hemorrhage were in the ≥ 60-year-old group.

These results show that comorbidity is higher in patients over 60 years of age (p<0.001). Possibly due to this, we think that the incidence of complications (p<0.001), hospitalization duration (p<0.001), time to begin oral feeding (p=0.001), and conversion to open surgery (p=0.034) increases in a statistically significant manner.

## Conclusions

The rate of surgery due to acute cholecystitis with a stone in the gall bladder, comorbid diseases, conversion to an open cholecystectomy, hospitalization period, the mean hour of initiation of oral nutrition, postoperative complication, and pulmonary complication were statistically significantly higher in the ≥60 years age group. In conclusion; laparoscopic cholecystectomy is a surgery that can be safely performed. However, it should be kept in mind that comorbidity may make the surgery and postoperative follow-up period complicated.
